# Genome Size, Molecular Phylogeny, and Evolutionary History of the Tribe Aquilarieae (Thymelaeaceae), the Natural Source of Agarwood

**DOI:** 10.3389/fpls.2018.00712

**Published:** 2018-05-29

**Authors:** Azman H. Farah, Shiou Yih Lee, Zhihui Gao, Tze Leong Yao, Maria Madon, Rozi Mohamed

**Affiliations:** ^1^Forest Biotech Laboratory, Department of Forest Management, Faculty of Forestry, Universiti Putra Malaysia (UPM Serdang), Seri Kembangan, Malaysia; ^2^Key Laboratory of Bioactive Substances and Resources Utilization of Chinese Herbal Medicine, Ministry of Education, Institute of Medicinal Plant Development, Chinese Academy of Medicinal Sciences and Peking Union Medical College, Beijing, China; ^3^Forest Research Institute Malaysia, Kuala Lumpur, Malaysia; ^4^Genomics Unit, Advanced Biotechnology and Breeding Centre, Malaysian Palm Oil Board, Kuala Lumpur, Malaysia

**Keywords:** *Aquilaria*, *Gyrinops*, flow cytometry, chloroplast genes, ITS gene

## Abstract

The tribe Aquilarieae of the family Thymelaeaceae consists of two genera, *Aquilaria* and *Gyrinops*, with a total of 30 species, distributed from northeast India, through southeast Asia and the south of China, to Papua New Guinea. They are an important botanical resource for fragrant agarwood, a prized product derived from injured or infected stems of these species. The aim of this study was to estimate the genome size of selected *Aquilaria* species and comprehend the evolutionary history of Aquilarieae speciation through molecular phylogeny. Five non-coding chloroplast DNA regions and a nuclear region were sequenced from 12 *Aquilaria* and three *Gyrinops* species. Phylogenetic trees constructed using combined chloroplast DNA sequences revealed relationships of the studied 15 members in Aquilarieae, while nuclear ribosomal DNA internal transcribed spacer (ITS) sequences showed a paraphyletic relationship between *Aquilaria* species from Indochina and Malesian. We exposed, for the first time, the estimated divergence time for Aquilarieae speciation, which was speculated to happen during the Miocene Epoch. The ancestral split and biogeographic pattern of studied species were discussed. Results showed no large variation in the 2C-values for the five *Aquilaria* species (1.35–2.23 pg). Further investigation into the genome size may provide additional information regarding ancestral traits and its evolution history.

## Introduction

Accommodating about 800 species worldwide, the family Thymelaeaceae was given its name by the French botanist Michel Adanson in 1763 (Adanson, 1763). At present, the family is divided into subfamilies Octolepidoidae and Thymelaeoideae, with the former having two groups, Gonystylus and Octolepis, and the latter having three tribes, Aquilarieae, Daphneae, and Synandrodaphneae (Herber, 2003). In general, Thymelaeaceae is a cosmopolitan family consisting of 45 genera, including two well-known agarwood-producing genera, *Aquilaria* Lam. and *Gyrinops* Gaertn., both in the tribe Aquilarieae. There are 21 accepted species in the genus *Aquilaria* and nine in the genus *Gyrinops* (The Plant List, 2013), of which 13 from *Aquilaria* (*A. baillonii, A. beccariana, A. crassna, A. filaria, A. hirta, A. khasiana, A. malaccensis, A. microcarpa, A. rostrata, A. rugosa, A. sinensis, A. subintegra*, and *A. yunnanensis*.) and five from *Gyrinops* (*G. caudata*, *G. ledermannii*, *G. salicifolia*, *G. versteegii*, and *G. walla*), reportedly produce agarwood (Mulyaningsih and Yamada, 2007; Lee and Mohamed, 2016b). In the natural environment, it takes several years for a wild tree to form agarwood after damage from a natural catastrophe, pest, or insect disturbance and microorganism colonization (Rasool and Mohamed, 2016). Some of the major uses of agarwood include its incorporation into perfumes, incenses, and traditional medicines. Although agarwood has been cultivated for at least two decades, the high demand for natural agarwood in the market continues to threaten the survival of wild populations. Due to overwhelming illegal harvesting, these species are currently protected and listed as endangered in Appendix II of the Convention on International Trade in Endangered Species of Wild Fauna and Flora (CITES, 2013).

Of the many agarwood species, only a few have been cultivated intensively, including *A. crassna, A. filaria, A. malaccensis, A. sinensis, A. subintegra*, and *G. versteegii*. However, basic information such as genome size and chromosome count, which are important for breeding efforts, are lacking for these non-model plant species. Generally, the chromosome number for the family Thymelaeaceae is reported as *x* = 9 (Herber, 2003), however, variations exist depending on the species. The chromosome count for *A. agallocha* (synonym *A. malaccensis*) has been determined as *x* = 8 (2*n* = 16) and the karyotype formula as 2*n* = 2*x* = 16 = 10m + 6sm (Debnath et al., 1995), where ‘*n’* is chromosome count, ‘*x*’ is the basic number, ‘*m*’ is metacentric, and ‘sm’ is submetacentric. There are two karyotypes for *A. sinensis* from China; the 2A type (2*n* = 2*x* = 16 = 10m +4sm + 2st) (Huang, 2009) is considered more primitive than the 2B type (2*n* = 16 = 6m + 6sm + 4st) (Shen et al., 2009), where ‘st’ is subtelocentric. From karyotype analysis, *A. agallocha* appears more primitive than *A. sinensis* (Zou et al., 2013). *Aquilaria* species are generally diploid, however, polyploids have been shown as possible through colchicine treatment *in vitro*. Naturally, *A. malaccensis* is 2*x* = 14, but induced tetraploids had double the chromosome number (4*x* = 28) (Siti Suhaila et al., 2015). There are also variations in genome size. For example, the genome size estimation for *A. agallocha* of Myanmar origin is 2*C* = 1.51 pg (Chen C.H. et al., 2014) while the estimation for *A. malaccensis* of Peninsular Malaysia is 2*C* = 1.86 ± 0.02 pg (Siti Suhaila et al., 2013). The estimation of *C*-value data for a certain plant family could contribute toward understanding the biological diversity significance and evolution of the family (Ng et al., 2017). Although the plant genome size database has been constantly increasing, information on genome size for tropical woody angiosperms is still lacking.

Several molecular phylogenetic studies have been carried out to investigate the broad scale genetic relationships in Thymelaeaceae. Molecular data from combined chloroplast locus *rbc*L and intergenic spacer region *trn*L-*trn*F are found insufficient to clarify relatedness of all the genera (Van der Bank et al., 2002). However, by adding the nuclear internal transcribed spacer (ITS) and multiplying the sampling size, Beaumont et al. (2009) increased the molecular data; the results support the taxonomy classification proposed by Herber (2003; Van der Bank et al., 2002). For its constituent genera in Thymelaeaceae, several molecular phylogenies have been reported: *Aquilaria* (Eurlings and Gravendeel, 2005; Lee and Mohamed, 2016a), *Dirca* (Schrader and Graves, 2004; Floden et al., 2009), *Gnidia* (Beaumont et al., 2009), *Gyrinops* (Eurlings and Gravendeel, 2005), *Lachnaea* (Robinson, 2008), *Passerina* (Van Niekerk, 2008), *Pimelea* (Motsi et al., 2010; Squire, 2016), *Thecanthes* (Motsi et al., 2010), and *Thymelaeae* (Galicia, 2006). Generally, *Gyrinops* is regarded as a sister to *Aquilaria*, and both genera are under the tribe Aquilarieae (Herber, 2003), nonetheless, its taxonomy category is still under debate. Some studies have suggested that *Gyrinops* should be synonymized with *Aquilaria*, because the sole delineating characteristic – the number of stamens – is not a strong taxonomic character (Eurlings and Gravendeel, 2005; Lee and Mohamed, 2016b).

Here, we constructed two phylogenetic trees from 15 different species in the tribe Aquilarieae, consisting of two genera, *Aquilaria* and *Gyrinops*, based on a combination of chloroplast DNA (cpDNA) *mat*K, *rbc*L, *trn*L intron, *trn*L-*trn*F and *psb*C-*trn*S sequences, and the nuclear ribosomal DNA (nrDNA) ITS region, and reported the genome size of five commonly available *Aquilaria* species using flow cytometry. Our analysis is the first that provides estimation on the time when genetic divergence occurred in the tribe Aquilarieae.

## Materials and Methods

### Genome Size Estimation

#### Plant Materials

Fresh young leaves from 5-year-old *Aquilaria* species: *A. hirta, A. malaccensis, A. microcarpa*, *A. sinensis*, and *A. subintegra*, were collected from lateral branches and immediately processed. Trees were grown in polybags in the plant nursery of the Faculty of Forestry, Universiti Putra Malaysia (UPM), Serdang, Malaysia. Identities of these species were authenticated in our previous studies (Lee and Mohamed, 2016a; Lee et al., 2016). A total of five pieces of leaves were collected from three individual plants to represent biological replicates of each species. For use as an external reference standard, oil palm (*Elaeis guineensis*, Arecaceae, 2*C* = 3.98 pg) (Madon et al., 2008) leaf sample was collected from trees growing in the arboretum of the Malaysian Palm Oil Board, Bandar Baru Bangi, Kajang, Malaysia. A 1 cm^2^ fragment was aseptically excised from the leaf using a sterile scalpel and directly transferred to prepare the nuclei for flow cytometry.

#### Preparation of Nuclei Suspension and Flow Cytometric Analysis

Using a sharp razor blade, approximately 1 cm^2^ of the leaf fragment was finely chopped in a Petri dish containing 1 mL of Otto I buffer (Dolezel et al., 2007). Subsequently, 500 μL of the same buffer was added and the suspension was filtered through a 40 μm nylon mesh and transferred into a Falcon tube. The nuclei suspension was mixed with 2 mL of Otto II buffer containing 1 μL of 50 μg mL^-1^ RNase A and 50 μm mL^-1^ propidium iodide (Dolezel et al., 2007). The stained nuclei suspension was then filtered through a 50 μm nylon mesh and incubated for 24 h at 4°C. The fluorescent nuclei were analyzed using a flow cytometer (FACSCalibur, Becton Dickinson) equipped with a 488 nm argon ion laser and 1,088 channels. The software CellQuest was used to measure nuclei images and the data was analyzed in the form of histogram peaks with a coefficient of variation (CV) generated from approximately 5,000 nuclei for each technical replicate. There were five technical replicates and three biological replicates for each species. Data collected included genome size values (2*C*) and the means were analyzed using an analysis of variance (ANOVA) and tested with Tukey HSD *post hoc* tests. *P* < 0.05 indicated significant differences.

### Molecular Analysis

#### Plant Materials

For sequence amplification, 15 species (**Table 3**) were selected based on prior knowledge of their identities. *A. beccariana*, *A. hirta, A. malaccensis*, and *A. rostrata* were part of our own field collection (Lee and Mohamed, 2016a; Lee et al., 2016, 2017), while other samples were donated as follows: *A. sinensis* and *A. yunnanensis* from Prof. Jianhe Wei [Institute of Medicinal Plant Development (IMPLAD), China], *A. crassna* and *A. subintegra* from Dr. Mohd Noor Mahat [Forest Research Institute Malaysia (FRIM), Malaysia], *A. rugosa* from Prof. Arunrat Chaveerach [Khon Kaen University (KKU), Thailand], *A. cumingiana*, *A. microcarpa, G. caudate*, and *G. versteegii* from Dr. Maman Turjaman [Forestry and Environment Research, Development and Innovation Agency (FOERDIA), Indonesia]. *A. agallocha* and *G. walla* were obtained from planted sources by traders.

Other plant materials were acquired as follows: *Gonystylus bancanus* and *Phaleria macrocarpa* were collected from a planted tree in the arboretum of the Faculty of Forestry, UPM. *Wikstroemia ridleyi* was collected as a plant specimen during a field expedition in Terengganu, Malaysia. *Aquilaria agallocha* is synonym to *A. malaccensis* at present, with a few morphological differences which were exempted. However, in order to aid our discussion, we treated *A. agallocha* and *A. malaccensis* separately, with the former confined to the northeast of the Indian continent and the latter to the Malesian region. Whenever possible, fresh leaves were used for genomic DNA extraction, while for donated samples, leaves were oven-dried at 60°C overnight, prior to transport. Voucher specimens were prepared and kept in the Forest Biotechnology Lab, Faculty of Forestry, UPM.

#### Genomic DNA Extraction, Polymerase Chain Reaction (PCR) Amplification and Sequencing

Genomic DNA was extracted using the Favorprep^TM^ Plant Genomic DNA Extraction Mini Kit (Favorgen, Taiwan) according the manufacturer’s suggested protocol. The isolated DNA was quantified using a Nanophotometer (Implen, Germany). A total of five chloroplast DNA (cpDNA) regions—two coding cpDNA loci, *mat*K and *rbc*L; the *trn*L intron; and two non-coding cpDNA intergenic spacer loci, *trn*L-*trn*F and *psb*C-*trn*S—and an nrDNA ITS region were amplified (**Table 2**). PCR was conducted in a final reaction volume of 25 μL, containing 12.5 μL of 2x PCRBIO Taq Mix Red (PCRBiosystems, United Kingdom), 10 mM of each primer, and 20 ng of genomic DNA as a template. PCR amplification was conducted in a MyCycler^TM^ thermal cycler system (Bio-Rad, United States). PCR conditions (Lee et al., 2016) and annealing temperatures for each primer set are shown in Supplementary Table S1. PCR products were visualized in 1% agarose gel prior to direct DNA sequencing (ABI PRISM 3730xl Genetic Analyzer, Applied Biosystems, United States), performed by the First Base Laboratory Sdn. Bhd., Malaysia. In the case where targeted sequences were available in the GenBank database through previous studies of the same plant specimen, the records were included in this study without performing additional PCR and sequencing (**Table 3**).

#### Phylogenetic Analyses

Five cpDNA and one nrDNA sequences from each species were edited to remove primer sequences, and then the cpDNA sequences were combined into a contiguous sequence in the order of *mat*K, *rbc*L, *trn*L intron, *trn*L-*trn*F, and *psb*C-*trn*S, resulting in a sequence 3,285–3,304 bp long; while the ITS sequence was 786–792 bp long. The length of the alignment generated using MUSCLE was 3,323 bp for the combined cpDNA sequences and 795 bp for the ITS sequence (Edgar, 2004). The number of indels and substitutions were calculated using MEGA 7 software (Kumar et al., 2016). A DNA substitution model, the jModelTest version 2.1.4 (Guindon and Gascuel, 2003; Darriba et al., 2012) was selected to assess our data set by implementing the hierarchical likelihood ratio test. According to the Akaike Information Criterion (AIC), the model best fitting the observed data for the combined cpDNA sequence was the general time reversible (GTR) model and invariable site (+I) (=GTR+I); while the nrDNA ITS sequence correlated best to the Tamura and Nei (TN93) model and gamma distributed (+G) (=TN93+G). Maximum likelihood (ML) trees were constructed using default parameters in MEGA 7, with 1,000 bootstrap replicates for each individual clade. Gaps and missing data were treated as complete deletions in this analysis. *Gonystylus bancanus*, *P. macrocarpa*, and *W. ridleyi* were used as an outgroup to root both trees before divergence times were estimated.

#### Calibration and Estimation of the Divergence Time of Aquilarieae

The evolution tree was constructed using the phylogenetic tree derived from the combined cpDNA sequences. There are no useful reports in the fossil evidence about Aquilarieae, therefore pairwise divergence time was conducted using fossil records from Cistaceae, a sister family that was recorded closest to Thymelaeaceae, using the TimeTree program (Hedges et al., 2006). The divergence time estimation was conducted using the Bayesian method implemented in the MCMCTREE v4.0 program in the PAML package (Yang, 2007). A Markov Chain Monte Carlo (MCMC) analysis was carried out based on the predicted divergence time calculated from the TimeTree program using the parameter: –rootage 102 –clock 2 –alpha 0.020000.

## Results

### 2*C* DNA Content

**FIGURE 1:**
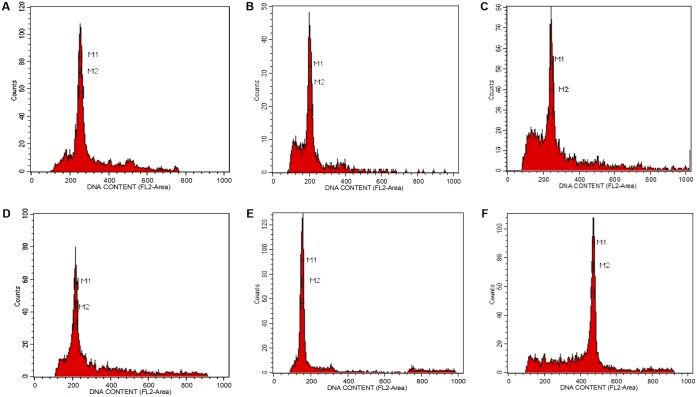
Fluorescence intensity histogram peaks showing the number of nuclei per channel as a function of relative fluorescence obtained after flow cytometric analysis of propidium iodide-stained nuclei. **(A)** *Aquilaria hirta*, **(B)** *Aquilaria malaccensis*, **(C)** *Aquilaria microcarpa*, **(D)** *Aquilaria sinensis*, **(E)** *Aquilaria subintegra*, and **(F)** *Elaeis guineensis*, which acted as an external reference standard.

The isolation of nuclei suspensions from five *Aquilaria* species was successful following the method delineated by Dolezel et al. (2007). The histogram of the fluorescence intensity outputs (**Figure 1**) had sharp peaks at the *G*_1_ stage of the cell cycle and the CV values were less than 3%. A CV of <5% is adequate, while a CV above this threshold suggests the presence of polyphenols, dried-silica, or debris contamination, making the sample unacceptable (Dolezel et al., 2007; Pellicer and Leitch, 2014). Generally, *Aquilaria* species are diploid (Siti Suhaila et al., 2013; Chen C.H. et al., 2014). The 2*C* genome size values for the selected *Aquilaria* spp. were in the range of 1.35–2.23 pg (**Table 1**). There was only a 1.65-fold difference between the largest genome size (*A. microcarpa*) and the smallest (*A. subintegra*). *Aquilaria malaccensis* and *A. sinensis* had significantly larger genome sizes than did *A. subintegra* (Tukey test, *P* < 0.05), while *A. microcarpa* and *A. hirta* had similar sized genomes. Based on this result, the selection of the tips of growing shoots in the self-renewing region of *Aquilaria* plants is suitable for flow cytometry analysis. It would be ideal to estimate the genome size for other species in the genus to have a better idea of the genome size variation, however, it was impossible to get specimens in the form of fresh young shoots for many of the species for this purpose, as many of the trees are not accessible or have unknown locations.

**Table 1:** Genome size of species used in this study and their closely related genera and families.

Family	Species	2*C* (pg) ± SD	1*C* (pg)	Reference
Thymelaeaceae	*Aquilaria agallocha*	2.5	0.98	Chen C.H. et al., 2014
	*Aquilaria hirta*	2.17 ± 0.061	1.09	This study
	*Aquilaria malaccensis*	1.86 ± 0.02	0.93	Siti Suhaila et al., 2013
	*Aquilaria malaccensis*	1.83 ± 0.028	0.92	This study
	*Aquilaria microcarpa*	2.23 ± 0.074	1.12	This study
	*Aquilaria sinensis*	1.87 ± 0.009	0.94	This study
	*Aquilaria subintegra*	1.35 ± 0.004	0.68	This study
	*Daphne alpina*	8.17 ± 0.10	4.09	Siljak-Yakovlev et al., 2010
	*Daphne blagayana*	4.70 ± 0.04	2.35	Siljak-Yakovlev et al., 2010
	*Daphne laureola*	4.70 ± 0.04	2.99	Siljak-Yakovlev et al., 2010
	*Daphne mezereum*	5.97 ± 0.06	3.03	Siljak-Yakovlev et al., 2010
	*Dirca palustris*	6.05 ± 0.01	3.03	Fridley and Craddock, 2015
	*Gnidia polystachya*	1.69 ± 0.35	0.85	Garcia et al., 2010
	*Pimelea linifolia*	7.46	3.73	Hanson et al., 2005
	*Thymelaea hirsuta*	3.29	1.65	Morgan and Westoby, 2005
Thymelaeaceae (15 records)			0.68-4.09	This study
Cistaceae (28 records)			0.88-4.50	Bennett and Leitch, 2012
Malvaceae (93 records)			0.19-4.10	Bennett and Leitch, 2012
Dipterocarpaceae (115 records)			0.267-0.705	Ng et al., 2017
Bixaceae (1 record)			0.18 ± 0.01	Bennett and Leitch, 2012


### Sequence Attributes

The entire alignment length for the 12 *Aquilaria* and three *Gyrinops* species contains 3,323 nucleotides of combined cpDNA sequences from *mat*K, *rbc*L, *trn*L intron, *trn*L-*trn*F and *psb*C-*trn*S fragments and 795 nucleotides from the ITS region. A total of 55 and 88 characters (1.66% and 11.07%) were variable informative; while 19 and 37 characters (0.57% and 4.65%) were parsimony informative, for the combined cpDNA and ITS region, respectively (**Table 2**). All sequences were deposited in the GenBank (**Table 3**). Occurrences of poly A/T sequence repetition were detected in the aligned *trn*L intron, *trn*L-*trn*F, and *psb*C-*trn*S sequences.

**Table 2:** Sequence attributes of the five loci used in this study.

	*mat*K	*rbc*L	*trn*L intron	*trn*L-*trn*F	*psb*C-*trn*S	ITS
Aligned length (bp)	834	624	587	469	826	795
Sequence length (bp)	828–834	617–624	555–583	447–463	757–816	786–792
Number of variable loci	54	10	46	41	48	88
Number of informative loci	8	2	5	9	4	37


**Table 3:** Localities, voucher details, and GenBank accession numbers of the selected species generated from this study, unless stated otherwise (refer to footnote).

Species	Collector’s name and collection number	Region of plant origin	GenBank accession numbers					
			
			*mat*K	*rbc*L	*trn*L intron	*trn*L-*trn*F	*psb*C-*trn*S	ITS
*Aquilaria agallocha*	Lee and Mohamed, JAI0001	Assam, India	MF443398	MF443405	MF443412	MF443428	MF443432	MH134137
*Aquilaria beccariana*	Lee et al., FBL04002	Johor, Malaysia	MF443399	MF443406	MF443413	MF443429	MF443433	MH134138
*Aquilaria crassna*	Lee and Mohamed, FBL01012	Vietnam	KU244186^a^	KU244212^b^	MF443414	KU244030^b^	MF443434	MH134139
*Aquilaria cumingiana*	Turjaman, MTJ0006	Maluku Islands, Indonesia	MF443400	MF443407	MF443415	KT726320	MF443435	MH134140
*Aquilaria hirta*	Lee and Mohamed, FBL01004	Terengganu, Malaysia	KU244190^b^	KU244216^b^	MF443416	KU244034^b^	MF443436	MH134141
*Aquilaria malaccensis*	Lee and Mohamed, FBL01001	Pahang, Malaysia	KU244193^b^	KU244219	MF443417	KU244037^a^	MF443437	MH134142
*Aquilaria microcarpa*	Lee and Mohamed, FBL01018	Kalimantan, Indonesia	KU244196^b^	KU244222^b^	MF443418	KU244040^b^	MF443438	MH134143
*Aquilaria rostrata*	Lee and Mohamed, FBL03001	Terengganu, Malaysia	MF443401	MF443408	MF443419	KT364475^a^	MF443439	MH134144
*Aquilaria rugosa*	Chuachan and Chaveerach, CC-AC974	Chiang Mai, Thailand	MF443402	MF443409	MF443420	MF443430	MF443440	MH134145
*Aquilaria sinensis*	Lee and Mohamed, FBL01021	Hainan, China	KU244202^b^	KU244228	MF443421	KU244046^b^	MF443441	MH134146
*Aquilaria subintegra*	Mohamed, FBL01015	Thailand	KU244205^b^	KU244231^b^	MF443422	KU244049^b^	MF443442	MH134147
*Aquilaria yunnanensis*	Lee and Mohamed, FBL01024	Yunnan, China	KU244207^b^	KU244233^b^	MF443423	KU244051^b^	MF443443	MH134148
*Gyrinops caudata*	Turjaman, MTJ0002	West Papua, Indonesia	MF443403	MF443410	MF443424	KT726323	MF443444	MH134149
*Gyrinops versteegii*	Lee and Mohamed, FBL01027	Lombok Island, Indonesia	KU244210^b^	KU244236^b^	MF443425	KU244054^b^	MF443445	MH134150
*Gyrinops walla*	Lee and Mohamed, FBL04005	Sri Lanka	MF443404	MF443411	MF443426	MF443431	MF443446	MH134151
*Gonystylus bancanus*	Lee, FBL01031	Selangor, Malaysia	KU244211^b^	KU244237^b^	MF443427	KU244055^b^	MF443447	MH135152
*Phaleria macrocarpa*	Lee, FBL04006	Indonesia	MH134155	MH134157	MH134159	MH134161	MH134163	MH134153
*Wikstroemia ridleyi*	Lee and Mohamed, FBL03010	Terengganu, Malaysia	MH134156	MH134158	MH134160	MH134162	MH134164	MH134154


*^*a*^Lee and Mohamed (2016a); ^*b*^Lee et al. (2016).*

### Aquilarieae Divergence Time Estimates Based on Five Combined Chloroplast Loci Sequences

**FIGURE 2:**
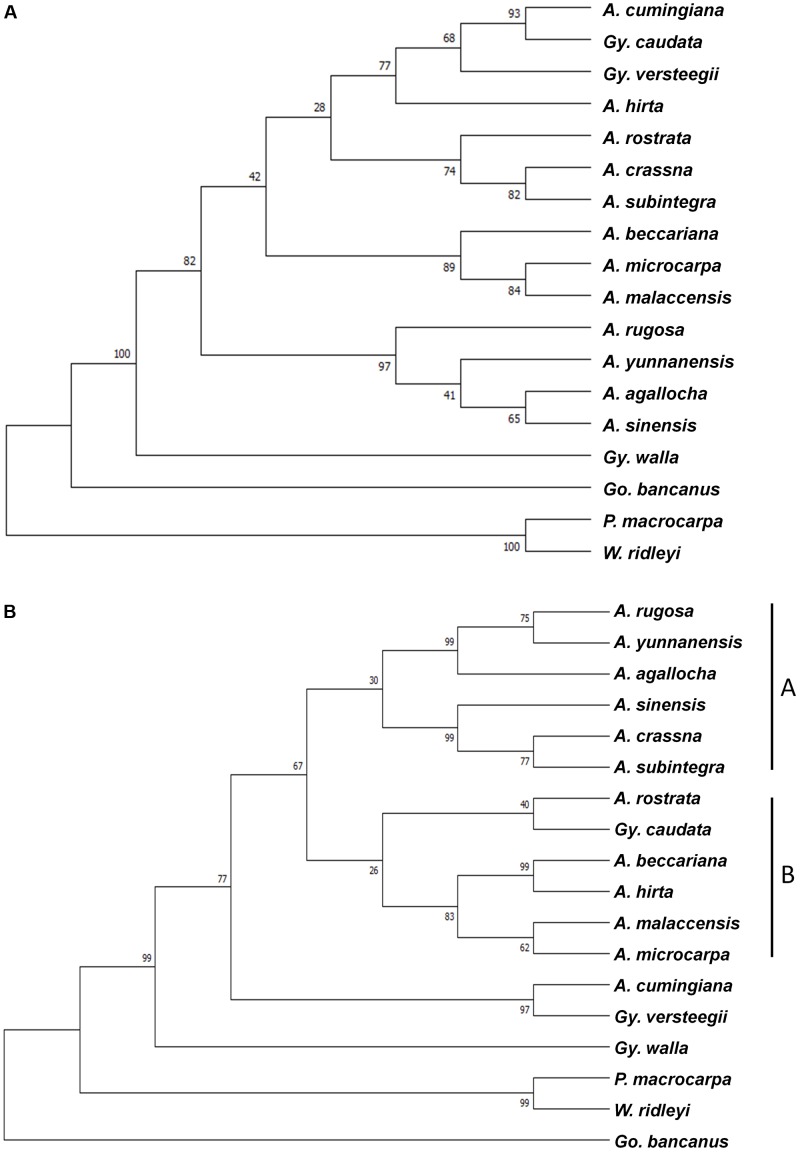
Maximum likelihood tree of 15 species from the Aquilarieae tribe based on **(A)** a combined data set of five chloroplast loci (*mat*K, *rbc*L, *trn*L intron, *trn*L-*trn*F, and *psb*C-*trn*S), and **(B)** the nuclear region DNA internal transcribed spacer (ITS) region. *Gonystylus bancanus*, *Phaleria macrocarpa*, and *Wikstroemia ridleyi* served as outgroups. Bootstrap values for a majority of divergences are >50%, as indicated on the corresponding nodes.

Combined sequences of the five chloroplast loci and the nrDNA ITS region were used to create the phylogenetic tree. Two methods, ML and Bayesian, were employed to acquire divergence time estimates for these species. For the combined cpDNA sequences, the jModeltest was used to identify the best substitution model for the mutation rate required for this analysis, which would further infer divergence times. The ML tree was constructed using MEGA 7, with 1,000 bootstrap replicates and a GTR+I model of substitution for the combined cpDNA sequences (**Figure 2A**), and a TN93+G model of substitution for the ITS region (**Figure 2B**). With the exception of *A. cumingiana*, the *Aquilaria* species were separated into two clusters in the ITS tree, in which clade A consisted of *A. agallocha*, *A. crassna*, *A. rugosa*, *A. sinensis*, *A. subintegra*, and *A. yunnanensis*, from the Indochina region; while clade B consisted of *A. beccariana*, *A. hirta*, *A. malaccensis*, *A. microcarpa*, and *A. rostrata*, from the Malesian region (**Figure 2B**). The branching between the Indochina and Malesian *Aquilaria* species showed moderate (67%) bootstrap support.

**FIGURE 3:**
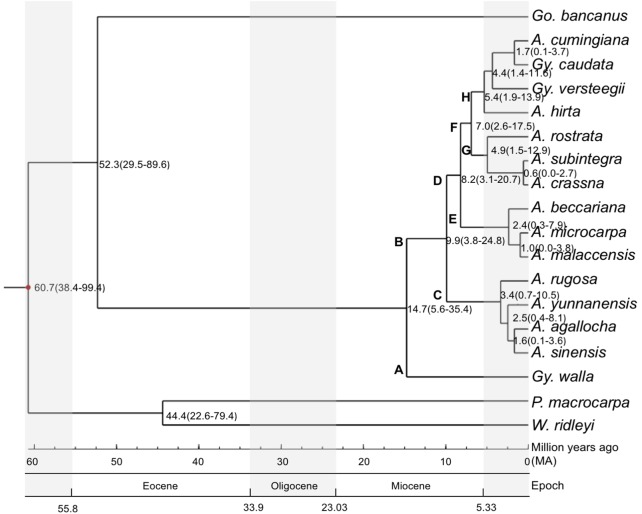
Divergence time estimation for species in the tribe Aquilarieae. Bayesian consensus tree inferred from the combined sequences of chloroplast non-coding genes *mat*K and *rbc*L, chloroplast gene intron *trn*L, and chloroplast intergenic spacer region *trn*L-*trn*F and *psb*C-*trn*S from 15 species in the Aquilarieae tribe. *Gonystylus bancanus*, *Phaleria macrocarpa*, and *Wikstroemia ridleyi* were used as outgroups. The red dot on the node represent estimated divergence time used to calibrate the analysis. The scales below the phylogenetic tree indicate the estimated divergence time in million years ago (Ma), which happened over several epochs. (A, *Aquilaria*; Gy, *Gyrinops*; Go, *Gonystylus*; P, *Phaleria*; W, *Wikstroemia*).

To estimate the divergence times of the branching of the phylogenetic tree, we used the estimated confidence interval (CI) provided by the TimeTree program (Hedges et al., 2006), between Cistaceae and Thymelaeaceae. Cistaceae served as the reference for the divergence time calibration. Ancestors of the three target species, *G. bancanus*, *A. malaccensis* and *G. walla*, had diverged at approximately the same time, 71 million years ago (Ma), with CI = 40–102 Ma and a median time of 80 Ma. The maximum value of the CI (102 Ma) was used as the anchor point for the calculations of the divergence times. **Figure 3** shows the estimated divergence time of the 15 studied species in Aquilarieae having a total of 11 clades. The first clade shows *P. macrocarpa* and *W. ridleyii* had split at 60.7 Ma (CI: 38.4–99.4 Ma). The two species then split to their respective clades at 44.4 Ma (CI: 22.6–79.4 Ma); while *G. bancanus* further split to form the fourth clade from other species of tribe Aquilarieae at 52.3 Ma (CI: 29.5–89.6 Ma). The fifth clade comprising *A. agallocha, A. rugosa, A. sinensis*, and *A. yunnanensis*, was formed at 9.9 Ma (CI: 3.8–24.8 Ma). Further speciation happened at 8.2 Ma (CI: 3.1–20.7 Ma) when *A. beccariana, A. malaccensis*, and *A. microcarpa* diverged from the fifth clade. The seventh clade (*A. crassna, A. rostrata*, and *A. subintegra*) diverged at 7.0 Ma (CI: 2.6–17.5 Ma), followed by the eight clade (*A. hirta*) at 5.4 Ma (CI: 1.9–13.9 Ma), and the ninth clade (*G. versteegii*) at 4.4 Ma (CI: 1.4–11.6 Ma). The last divergence for the tenth (*G. caudata*) and eleventh (*A. cumingiana*) clades happened quite recently at 1.7 Ma (CI: 0.1–5.7 Ma). The latest divergence that happened in Aquilarieae as found in this study was between *A. malaccensis* and *A. microcarpa* at 1.0 Ma (CI: 0.0–3.8 Ma), and between *A. crassna* and *A. subintegra* at 0.6 Ma (CI: 0.0–2.7 Ma).

## Discussion

As information about the *Aquilaria* genome size is still lacking, we conducted genome size estimations for selected species commonly used in plantation to provide ground base information for this genus. Genome size or *C*-value refers to the amount of DNA in a cell nucleus whether in an unreplicated monoploid chromosome set (*n*) or in a polyploid nucleus (Leitch and Leitch, 2013). Genome size has been shown to have a significant influence on plant ecophysiology and biodiversity (Greilhuber and Leitch, 2013). The genome sizes of several species in Thymelaeaceae, with the exception of *Aquilaria*, such as from the genera *Daphne*, *Dirca*, *Gnidia*, *Pimelea*, and *Thymelaea*, have been reported (**Table 1**). Generally, *Aquilaria* species have a genome size in the range of 2*C* = 1.35–2.5 pg, while other species from the same family, namely *Daphne alpina*, *D. blagayana*, *D. laureola*, *D. mezereum*, *Dirca palustris*, *Gnidia polystachya*, *Pimelea linifolia*, and *Thymelaea hirsuta*, have genome sizes between 1.69 and 8.17 pg (Hanson et al., 2005; Morgan and Westoby, 2005; Garcia et al., 2010; Siljak-Yakovlev et al., 2010; Fridley and Craddock, 2015). Thus, *Aquilaria* species have a smaller genome size than other group members like *Daphne*, *Dirca*, *Pimelea*, and *Thymelaea*, but no clear pattern was found through this alignment within the same family. Genome size in plants could be an important indicator for their growth behavior; for example, plants with a smaller genome size exhibit faster canopy growth when compared to plants with a larger genome size (Siljak-Yakovlev et al., 2010), suggesting that the smaller genome might allow for faster growth due to faster cell division in the plant itself. From our personal observations in the field and through a review of the literature, *A. subintegra* can be regarded as a fast-growing species while *A. hirta* is slow-growing, relative to other commonly cultivated *Aquilaria* species (Lee and Mohamed, 2016b). In contrast, when comparing genome sizes of close families within the same order Malvales, the genome size of Thymelaeaceae is smaller (median 1*C* = 1.73 pg; 0.68–4.09, 15 records) than that of Cistaceae (median 1*C* = 2.40 pg; 0.88–4.50 pg, 28 records) and Malvaceae (median 1*C* = 1.39 pg; 0.19–4.10 pg, 93 records), but greater than that of Dipterocarpaceae (median 1*C* = 0.417; 0.267–0.705 pg, 115 records) (Bennett and Leitch, 2012; Ng et al., 2017). Based on this information, our findings were consistent with those of Ohri (2005) and Chen S.C. et al. (2014), at least at the family level, suggesting that woody angiosperms may possess smaller genome sizes and rarely exhibit polyploidism.

To obtain first-hand preliminary information on the evolution pattern of the Aquilarieae tribe, we selected five different chloroplast loci and combined their sequences to construct a phylogenetic tree consisting of 15 species from the tribe (**Figure 2**). Although it is advisable to include more loci in the tree, based on our experiences, we found that some of the chloroplast loci may display little or no nucleotide variations between different species within a genus. This is especially true for *Aquilaria*, when using gene markers such as *rpo*B, *rpo*C1, and *psb*A-*trn*H (Lee et al., 2016). In phylogeny studies, a high percentage of polymorphic sites is requisite to obtain useful information on the genetic distance between different species. The sequences amplified by the five selected primer sets in this study contain a number of polymorphic sites, which were helpful for the phylogenetic analysis. However, as these species are closely related via taxonomy, the percentage of conserved sites may be high relative to taxonomically distant species. For a better resolution and thorough information on the evolution analysis of a studied genus, the full chloroplast genome sequences is commonly utilized to calculate the divergence times of its taxa and to draw comprehensive conclusions and inferences regarding taxonomical aspects, genetic diversity, and the pattern of evolution of the studied species (Yang et al., 2013; Daniell et al., 2016). The only two published full chloroplast genome sequences of Aquilarieae species is of *A. sinensis* (Wang et al., 2016) and *A. yunnanensis* (Zhang et al., 2018); while a complete chloroplast genome record of *Daphne kiusiana* (Cho et al., 2017) and an unpublished partial chloroplast genome of *G. bancanus* (EU849490) in the NCBI GenBank database are the third and fourth references available for the family Thymelaeaceae. Without a concerted effort from various parties to sequence as many species of diverse origins as possible, the ability to sequence a complete genome by one research group requires unobtainable resources. Thus, we resorted to sequencing multiple chloroplast loci.

Taxonomy of the Aquilarieae tribe is still inconclusive. An attempt to resolve its taxonomical standing by relying on phylogenetic observations is beyond the scope of this study. Yet some suggestions for changes are worth highlighting in order to clarify relationships during future revisions. Although the cpDNA ML tree in general indicates a less powerful discrimination, out of the 13 nodes, two had bootstrap support values ≥95%, four have bootstrap support values between 75% and 94%, and seven have <75% (**Figure 2**). From general observation, the branching of the cpDNA ML tree does not reveal a clear pattern of the grouping. Eurlings and Gravendeel (2005) reported the paraphyletic placement of *Aquilaria* and *Gyrinops* using a single chloroplast locus, *trn*L-*trn*F. In this study, with the addition of four chloroplast loci sequences, the two genera in the Aquilarieae tribe appeared to show a different relationship. The type species *A. malaccensis* and *G. walla*, instead of forming two separate clusters comprising all species of the same genus, were, instead, traced to a common ancestral origin of both genera. The suggestion to reduce *Gyrinops* to *Aquilaria* synonym was again brought forward to reflect the natural resemblances among agarwood-producing species (Eurlings and Gravendeel, 2005; Lee and Mohamed, 2016b). This controversy had been raised since 1922, when the unification of the two genera was advocated as they share many similarities in morphological characteristics (Hallier, 1922; Hou, 1960).

The use of the nrDNA ITS region to gather useful genetic information has revolutionized plant phylogenetic studies at the species level for the last two decades due to its biparental inheritance that can be used to assess phylogeny at family and higher levels (Baldwin, 1992). The ITS region has a tendency to be homogenized for its sequence variation in concerted evolution and is easily amplified in the majority of plant species. These properties have caused the ITS region to gain wide popularity in modern plant phylogenetic studies (Neves and Forrest, 2011). The first report to utilize ITS to evaluate phylogeny relationships at the nuclear level among agarwood-producing species was carried out by Lee and Mohamed (2016a), and comprised seven *Aquilaria* species of Malaysia and Thailand origins, followed by a phylogeny study on the genetic diversity of eight different species from *Aquilaria* and *Gyrinops* from Indonesia (Lee et al., 2018). In this study, the ITS analysis was less congruent with the combined cpDNA analysis, however, both had similar amounts of strong bootstrap supports (≥75%, 9/15 in combined cpDNA analysis; 10/15 in ITS analysis) although the informative sequence variation was likely to be higher in the ITS region than in the combined cpDNA sequences. However, studies have shown that data from the ITS region could indicate that biogeographical information of a large genus is separated into clades (Fritsch, 2001; Li et al., 2010). Based on our own hypothesis, at least in the case of the genus *Aquilaria* (excluding *A. cumingiana*), the non-monophyletic relationship may be related to biogeographical factors, because separation of the two clades is in agreement with their regions of origin (Indochina and Malesia). The latest taxonomy revision for Thymelaeaceae proposed two major sub-families: Octolepidoidae and Thymelaeoideae (Herber, 2002), supported by molecular phylogenetic analysis using combined cpDNA loci, *rbcL* and *trn*L-*trn*F, and the nrDNA ITS (Beaumont et al., 2009). The phylogenetic relationship shows the sub-family Octolepidoidae (which includes *Gonystylus*) of having ancestral ties to Thymelaeoideae, while the tribe Synandrodaphneae has ancestral ties to Aquilarieae and Daphneae (which includes *Phaleria* and *Wikstroemia*). The same displacement was observed in our ITS tree (**Figure 2B**), but not in the combined cpDNA tree (**Figure 2A**). This could be explained with the first molecular phylogeny on Thymelaeaceae carried out only with the combined cpDNA of the *rbc*L gene and *trn*L-*trn*F, returned with an inaccurate classification at tribe level in Thymelaeaceae (Van der Bank et al., 2002). The inclusion of an additional ITS region with the former two cpDNA loci had fully resolved the latest taxonomy displacement proposed for Thymelaeaceae. Explanations based on the genetic information from the ITS region in this family plays a major role in constructing a matching result for the phylogenetic tree to the proposed classical taxonomical system. Considering that molecular phylogenetics in Thymelaeaceae is well-resolved at the tribe level, our study analyzed the two nucleotide loci separately, as they are from two different inheritance systems.

Another interesting finding is the dispersing clade of *A. agallocha* that originated from India and its synonym *A. malaccensis* from the Malay Peninsula (**Figures 2A,B**) (The Plant List, 2013). While identification of these species is primarily based on conventional techniques relying mostly on their morphological characteristics, the sequences of the same species from different geographical regions may contain variations. Phenotypic changes due to geographical boundaries, which function as buffer zones that prevent out-crossings between natural populations, may be one of the few possible factors leading to genetic variation in the natural population of *A. malaccensis*, since their intra-specific genetic variation can be larger than inter-specific genetic variation (Lee et al., 2017). To synonym *A. agallocha* with *A. malaccensis* was first proposed in 1836 (Society for the Diffusion of Useful Knowledge, 1838). However, Ridley (1901) observed that *A. agallocha*, when compared to *A. malaccensis*, had: (1) bigger size, (2) leaves with more veins, (3) a twofold to fourfold higher number of flowers per umbel (*A. malaccensis* has 10 flowers per umbel), (4) solitary umbels (*A. malaccensis* has panicled umbels), (5) larger flowers, and several other differences. Both tree species can be differentiated based on their morphological characteristics, even if their fruits may look alike. In contrast, Hou (1964) was inclined to synonym the two species, categorically classifying the morphological differences as exemptions. To aid our discussion, we treated *A. agallocha* and *A. malaccensis* separately, with the former confined to the northeastern section of the Indian continent and the latter to the Malesian region. We acknowledge that a detailed taxonomic revision should be undertaken by plant taxonomists to resolve these species names.

In common practice, useful references for divergence time calibrations are based on fossil evidence. Our searches for recorded fossil evidence for Thymelaeaceae in the Paleobiology database^1^ returned 10 records, of which only one was old, dated during the Miocene period (23.0–2.6 Ma), while the remaining nine specimens were quite recent, dated in Early Pleistocene (2.6–0.8 Ma). Based on the review by Herber (2003), the oldest fossil evidence for Thymelaeaceae was recorded from the Eocene (Venkatachala and Kar, 1969), which was closely related to *Gonystylus* (Venkatachala et al., 1988). Additional fossil records in pollen form, which dated to the Oligocene and Miocene (Muller, 1972; Anderson and Muller, 1975), also resemble *Gonystylus*. Unfortunately, we could not locate fossil evidence for specimens in the tribe Aquilarieae. As an alternative to fossil evidence, we used the estimated confidence interval (CI) provided by the TimeTree program to estimate the divergence times of the species, as indicated on the branching nodes of the phylogenetic tree (**Figure 3**). We selected the sister family Cistaceae as the reference specimen to conduct pairwise divergence time analyses targeting the tribe Aquilarieae since the paleobotany of this species is well documented (Guzman and Vargas, 2009). Although there are many possible molecular clock estimations of branching times errors (Sanderson et al., 2004), we obtained branching times through integrative methods, which utilized all present information from published literatures and compiled databases. We concluded that the estimated divergence time tree is considerably reliable, as supported by splitting of *G. bancanus* at 52.3 Ma, during the Eocene (55.8–33.9 Ma) age, and this complements the oldest fossil recorded from India, which resembles *Gonystylus* (Venkatachala and Kar, 1969).

We deduced the biogeographic pattern in the speciation of the tribe Aquilarieae by observing the estimated divergence time tree (**Figure 3**). It can be postulated that Aquilarieae had diverged during the Miocene (23.03–5.33 Ma) age. This assumption is based on the earliest estimated origin of Aquilarieae at 14.7 Ma, from which the first lineage A gave rise to *G. walla*, currently confined to Sri Lanka and the extremity of southwest India (Subasinghe et al., 2012), and to lineage B, presently distributed in northern India, Indochina, and Malesia. Lineage C diverged into the extant species now primarily confined to mainland Asia, ranging from northeast India (*A. agallocha*), southern China and Indochina (*A. sinensis*, *A. yunnanensis*, and *A. rugosa*). Lineage D gave rise to the Southeast Asian species. It further diversified into lineages E and F, with the extant species of lineage E confined to west Malesia and lineage F diversified into lineages G and H. Most of the species included in the current study had diversified during the Pliocene (5.33–2.58 Ma) and Pleistocene (2.58–0.01 Ma), when intensification of icehouse condition was present and much of it persisted through Pleistocene (Corner, 1960). Extant species of lineage G are confined to Indochina, Thailand and northern Malay Peninsula. Apparently, the narrowly distributed endemics, *A. subintegra* and *A. rostrata* had arose from allopatric speciation. Lineage H diversified in the Malay Archipelago (*A. hirta*), and nearby regions such as Celebes, Lesser Sunda Islands (*G. versteegii*), Moluccas Archipelago (*A. cumingiana*) and the Sahul Shelf (*G. caudata*).

*Aquilaria beccariana* and *A. hirta* display a disjunctive distribution across the South China Sea. *Aquilaria beccariana* is predominant north and east of Borneo Island, with a small population found in the southeastern Malay Peninsula. *Aquilaria hirta* is a swamp species widely distributed in the eastern and southern Malay Peninsula, western Kalimantan, and larger islands of the Riau Archipelago. These two species were once probably more widely distributed but retracted into the humid forest refuge known as Riau Pocket (Corner, 1960; Ashton, 1992, 1995, 2014) during the dry Last Glacial Maximum that affected Sundaland (Cannon et al., 2009).

This is the first account attempting to explain the biogeographic pattern of Aquilarieae using time-calibrated phylogeny. Fossil calibration, if available, would increase the accuracy of our postulation. We demonstrated the use of flow cytometry techniques to estimate the genome size of five *Aquilaria* species, which are found to be significantly smaller than those of other genera in the same family. Future investigation into the correlation between genome sizes in the tribe Aquilarieae based on the two different genera may provide further information into ancestral traits and association in their evolutionary history. Moreover, we used ITS analysis to show that *Aquilaria* is non-monophyletic. Using a chloroplast gene-based phylogenetic tree, we described the relationship and divergence times between the species in the tribe Aquilarieae. It will be highly interesting to re-analyze this data using chloroplast genome phylogeny, to recalculate the divergence times, and to address the differences, if any, in order to test the robustness of what we put forth here as our postulation in speciation and biogeographic of these species. We expect that information obtained from this work can help clarify the genetic relationship between the two genera in the tribe Aquilarieae, as well as to provide a preliminary view of the ancestral time divergence pattern within the tribe.

## Data Availability

The datasets generated or analyzed during this study can be found in the GenBank, accession codes MF443398–MF44344 and MH134137–MH134164, and in the Supplementary Information for this article.

## Author Contributions

RM and SL designed the study. AF and SL performed the experiments, led the writing with contributions from ZG and TY, and analyzed the data with help from ZG and TY. ZG provided the analyses for phylogeny and divergence time. MM aided in the cytometry experiment. RM reviewed and edited the manuscript. All authors reviewed and approved the final manuscript.

## Conflict of Interest Statement

The authors declare that the research was conducted in the absence of any commercial or financial relationships that could be construed as a potential conflict of interest. The reviewer QF and handling Editor declared their shared affiliation.

## Footnotes

1 www.paleobiodb.org
