# Supplementary material for: Genome Size, Molecular Phylogeny, and Evolutionary History of the Tribe Aquilarieae (Thymelaeaceae), the Natural Source of Agarwood
Source: Front Plant Sci. 2018 May 29;9:712. doi: 10.3389/fpls.2018.00712 (PMC5987174; doi:10.3389/fpls.2018.00712)
Supplement: Supplementary file 1 [file Table_1.docx]

**Genome size, molecular phylogeny and evolutionary history of the tribe Aquilarieae (Thymelaeaceae), the natural source of agarwood**

Hanani Azman Farah, Shiou Yih Lee, Zhihui Gao, Tze Leong Yao, Maria Madon and Rozi Mohamed

**Supplementary Table S1.** Details on the PCR primers and annealing temperatures used in this study

| Gene | Primer name | Sequence (5’-3’) | Annealing temperature (°C) | Reference |
| --- | --- | --- | --- | --- |
| *mat*K | KIM 1R | ACCCAGTCCATCTGGAAATCTTGGTTC | 53 | Kim K., Korea University, Seoul, Korea, unpubl. res. |
|  | KIM 3F | CGTACAGTACTTTTGTGTTACGAG |  | Kim K., Korea University, Seoul, Korea, unpubl. res. |
| *rbc*L | aF | ATGTCACCACAAACAGAGACTAAAGC | 55 | Kress and Erickson, 2007 |
|  | aR | CTTCTGCTACAAATAAGAATCGATCTC |  | Kress and Erickson, 2007 |
| *trn*L intron | c | CGAATCGGTAGACGCTACG | 50 | Taberlet et al., 1991 |
|  | d | GGGGATAGAGGGACTTGAAC |  | Taberlet et al., 1991 |
| *trn*L-*trn*F | e | GGTTCAAGTCCCTCTATCCC | 55 | Taberlet et al., 1991 |
|  | f | ATTTGAACTGGTGACACGAG |  | Taberlet et al., 1991 |
| *psb*C-*trn*S | CS2_F | GTTTTACGGGCCCACTGGAC | 50 | This study |
|  | *trn*S | GGTTCGAATCCCTCTCTCTC |  | Demesure et al., 1995 |
| ITS | ITS92 | AAGGTTTCCGTAGGTGAAC | 55 | Baldwin, 1992 |
|  | ITS-S3R | GACGCTTCTCCAGACTACAAT |  | Chen et al., 2010 |
